# Beliefs about causes of cancer among students around the world

**DOI:** 10.3389/fonc.2025.1631997

**Published:** 2025-08-01

**Authors:** Judyta Bordakiewicz, Donata Pokorska, Daria Cieniawa, Mateusz Mikołajczyk, Anita Mikołajczyk, Monika Rucińska, Karolina Osowiecka

**Affiliations:** ^1^ Student Scientific Circle Epidemiological Research, School of Public Health, University of Warmia and Mazury in Olsztyn, Olsztyn, Poland; ^2^ Faculty of Medicine and Dentistry, Medical University of Warsaw, Warsaw, Poland; ^3^ Department of Psychology and Sociology of Health and Public Health, School of Public Health, University of Warmia and Mazury in Olsztyn, Olsztyn, Poland; ^4^ Department of Oncology, Collegium Medicum, University of Warmia and Mazury in Olsztyn, Olsztyn, Poland

**Keywords:** cancer risk factors, genetic factors, lifestyle, cancer prevention, students

## Abstract

**Introduction:**

Early prevention, especially among young adolescence, could reduce cancer incidence. The aim of the study was to assess beliefs on cancer risk factors among students.

**Material and methods:**

The study was conducted among 761 Polish students and 140 students from 32 other countries. The study was provided using an original, validated questionnaire.

**Results:**

The most commonly indicated cancer risk factors by both Polish and foreign students were smoking cigarettes, drinking alcohol, sunbathing/using tanning beds, exposure to ionizing radiation, diet, and exposure to harmful occupational factors. There is a gap in knowledge concerning some other risk factors: low physical activity, viral infection, and hormonal contraception. Students indicated genetic factors and smoking as a main cancer causes. Medical students were more aware of different cancer risk factors compared with non-medical students. Nationality (Polish/foreign), medical field of study, female, having cancer patient among friends/family, and living in bigger city were significantly associated with beliefs about different cancer risk factors.

**Conclusion:**

Medical students demonstrated better knowledge about cancer causes than non-medical students, but it seems that the genetic factor is overestimated by students. It is necessary to provide education even among teenagers to increase cancer prevention. Special attention in raising awareness should be paid to cancer risk factors of physical inactivity and viral infection.

## 1 Introduction

Every year, about 10 million people worldwide die from cancer (1). Cancer affects not only the length of life but also its quality (2). Globally, cancer has become an expanding health, economic, and social burden (3). The most recent report from the Polish Cancer Registry, published in 2023, indicates that approximately 1.17 million individuals in Poland have currently diagnosis of cancer, and around 100,000 patients die from cancer each year (4). Cancer is the second leading cause of mortality in Poland, after cardiovascular disorders, accounting for 25% of all deaths (5).

Public awareness of potential causes of cancer, screening tests and other activities that can reduce the risk of disease is insufficient (6). Lack of knowledge induces cancer-promoting behavior and an inability to avoid risk factors, whereas 44% of cancer deaths are due to modifiable variables (7), which are common in all developed countries (8). Modifiable factors are, for instance, tobacco smoking, obesity and diet (processed meat and lack of fiber), infections, sun exposure, alcohol drinking, and hormonal contraception (9). Recent studies show that Polish citizens have a lower tendency to attend cancer screenings than other European countries (1). It has been proven that Polish women present a low level of recognition of both the risk factors and importance of screening for namely breast cancer (10).

Early prevention has a chance of reducing the possibility of cancer in the future (11). Therefore, knowledge about cancer should be available not only to older people, among whom cancer is most common, but especially to young people to allow them to induce changes in their lifestyle or obtain an early diagnosis to decrease cancer morbidity and mortality rates in Poland and worldwide. Students’ education on the subject is extremely important, as young adults typically begin engaging in unhealthy habits, such as alcohol consumption or smoking, during their college years (12).

The significance of modifiable risk factors was outlined as early as 1974 in the “New Perspective” report, which introduces the concept of “health field” according to which four interdependent determinants constitute a base for properly functioning healthcare systems among which lifestyle accounts for 50% (13). The World Health Organization states that between 30% and 50% cancer deaths could be prevented by lifestyle modifications or avoidance of principal cancer contributors with an additional implementation of existing evidence-based prevention strategies (14). Based on that, the shift of the focus point from tertiary care to primary prevention appears to be more beneficial, as a large percentage of cancer types could be prevented instead of treated.

The results of previous studies conducted on students showed a lack of sufficient knowledge about cancer and suggested the need for appropriate education (15–17). Young people not only do present inadequate knowledge of cancer causative factors but also tend to believe in discredited information.

Objective of the study was to assess beliefs related to the causes of cancer among medical and non-medical students enrolled in various university courses around the world.

## 2 Materials and methods

### 2.1 Participants

The survey involved 901 students, including 761 Polish students and 140 students of 32 nations from various universities around the world. The study was carried out between December 2023 and February 2025.

The survey was planned on a group of 832 students (416 Polish students and 416 foreign students) to be representative for the cross-sectional study. Access to the online survey was open, and there were more than planned number of responses. Unfortunately, the discrepancy between Polish and foreign students was noted.

The selection criteria included university student status. The medicine students of third year and above were excluded.

### 2.2 Questionnaire design

The study was carried out using a questionnaire designed specifically for this study in accordance with general principles. The questionnaire was a simple tool, consisted of six main closed-ended quantitative questions. The questionnaire included four single-choice questions (nominal scale: yes, no, and I do not know) and two multiple-choice questions (with the possibility for choosing potential cancer risk factors from a list of 31 options and maximum of three main potential cancer risk factors). The questionnaire was supplemented by eight questions concerning sociodemographic data (age, gender, country and place of residence, nationality, field of study, year of study, and occurrence of cancer in family or close friends). The questionnaire was designed in cooperation with an oncologist, a psycho-oncologist, and a public health specialist.

#### 2.2.1 Comprehensibility and acceptability

The comprehensibility and acceptability analysis was conducted on a group of 23 Polish students of the medical faculty. Students filled out the main questionnaire and the validation procedure questionnaire. Information about the time taken to complete the questionnaire and about the structure, form and clarity, comprehensibility, and suitability of the questionnaire was collected. The mean time taken for completion of the questionnaire was 3 ± 1.3 min (ranged 1–5 min). All participants considered that format of the questionnaire was good and the font size was big enough. Participants claimed that the questionnaire was sufficiently long (except two students, who indicated that survey might be longer). For all participants, questions were understandable and there were no questions that they did not want to provide an answer. Four respondents (17%) indicated that the questionnaire’s items had noted that someone might consider the punishment of sins, lack of luck, or a matter of chance as a risk factor for cancer.

#### 2.2.2 Reliability

The reliability analysis was performed on a group of 69 medical students and included comparison of two sets of responses to questions from the same student within a 2-week interval. In the analysis of the repeatability of answers to the questions, the percentage agreement was calculated. Cohen’s Kappa coefficient was used to assess the agreement of two measurements of a variable. The criteria for compliance according to Landis and Koch were as follows: 0–0.20, slight agreement; 0.21–0.40, fair agreement; 0.41–0.60, moderate agreement; 0.61–0.80, substantial agreement; and 0.81–1.00, almost perfect agreement. The percentage of consensus responses for multi-choice questions was 78%–100% and for single-choice questions was 96%–100%. Test-retest reliability coefficients ranged from 0.74 to 1.00 over a 2-week interval.

#### 2.2.3 Questionnaire translation

The English version of the questionnaire was developed by a back-translation procedure. The Polish version of the questionnaire was translated into an English version by two independent professional translators. The English version was then translated back into Polish by another translator. The discrepancies from this back-translation were consulted with a psycho-oncologist and a public health specialist, and adequate modifications were introduced. Polish and English versions of questionnaire are presented in **Supplementary Materials**.

### 2.3 Data collection

The data were collected using questionnaire in paper form or online via the Google Forms. Students of Student Scientific Circle Epidemiological Research at University of Warmia and Mazury in Olsztyn in Poland distributed the questionnaire to various Polish and foreign universities and were responsible for coordinating the acquisition of data. Participation in the study was voluntary and anonymous. By starting to fill in the survey, thee respondents gave their consent to participation in the study.

### 2.4 Statistical analysis

The chi-square test was used to compare the proportions of beliefs about different causes of cancer between Polish vs. foreign students and medical vs. non-medical students. Multivariate analysis was conducted using multiple logistic regression analyses. Logistic regression models with the backward elimination method were performed to determine the relationship between students’ beliefs about different causes of cancer and a set of independent variables (i.e., gender, nationality, place of residence, field of study, and occurrence of cancer in a close friend/family). The odds ratio (OR) with a 95% confidence interval (CI) was calculated for categories: “yes” vs. “no.” A p-value of <0.05 was considered to be significant. The data analysis was conducted using Statistica (data analysis software), version 13 [StatSoft Polska Sp. z o.o., 2024; www.statsoft.pl].

### 2.5 Ethical agreement

The study was conducted in accordance with the Declaration of Helsinki and approved by the Ethics Committee of the University of Warmia and Mazury in Olsztyn (No. 34/2023; 30 November 2023).

## 3 Results

### 3.1 Characteristics of participants

The study included 901 participants: 761 Polish students and 140 foreign students from 32 nationalities. Foreign students’ group was dominated by the following: Indians (19%), Swedish (9%), Italian (9%), Pakistani (7%), Spanish (6%), German (5%), Iraqi (5%), Mexican (5%), Norwegian (5%), French (4%), Japanese (3%), Turkish (3%), Danish (2%), Filipino (2%), and Romanian (2%) (Table A in the **Supplementary Materials**). Respondents were aged 18–26 years (median age, 20 years for students from Poland and 23 years for students from other countries). The majority of students were women, lived in big cities, and had a relative or friend diagnosed with cancer. Medical students (as defined by students of medicine, nursing, midwifery, emergency medicine, rehabilitation, and dietetics) accounted for 46% of Polish participants and 35% of foreign participants (**Table 1**).

**Table 1:** Characteristics of study group.

Variables	Participants			
	Polish students n=761		Foreign students n=140	
	N	%	N	%
Sex				
women	555	72.9	93	66.4
men	177	23.3	47	33.6
no data	29	3.8	0	0.0
Place of residence				
village	250	32.8	8	5.7
cities < 50 thousands inhabitants	155	20.4	15	10.7
cities 50-100 thousands inhabitants	71	9.3	13	9.3
cities >100 thousands inhabitants	260	34.2	104	74.3
no data	25	3.3	0	0.0
Field of study				
medical	352	46.3	49	35.0
non-medical	409	53.7	87	62.1
no data	0	0.0	4	2.9
Having cancer patient as a close friend/family				
yes	530	69.6	70	50.0
no	184	24.2	61	43.6
I do not know	45	5.9	9	6.4
no data	2	0.3	0	0.0

### 3.2 Risk factors of cancer

**Figure 1:**
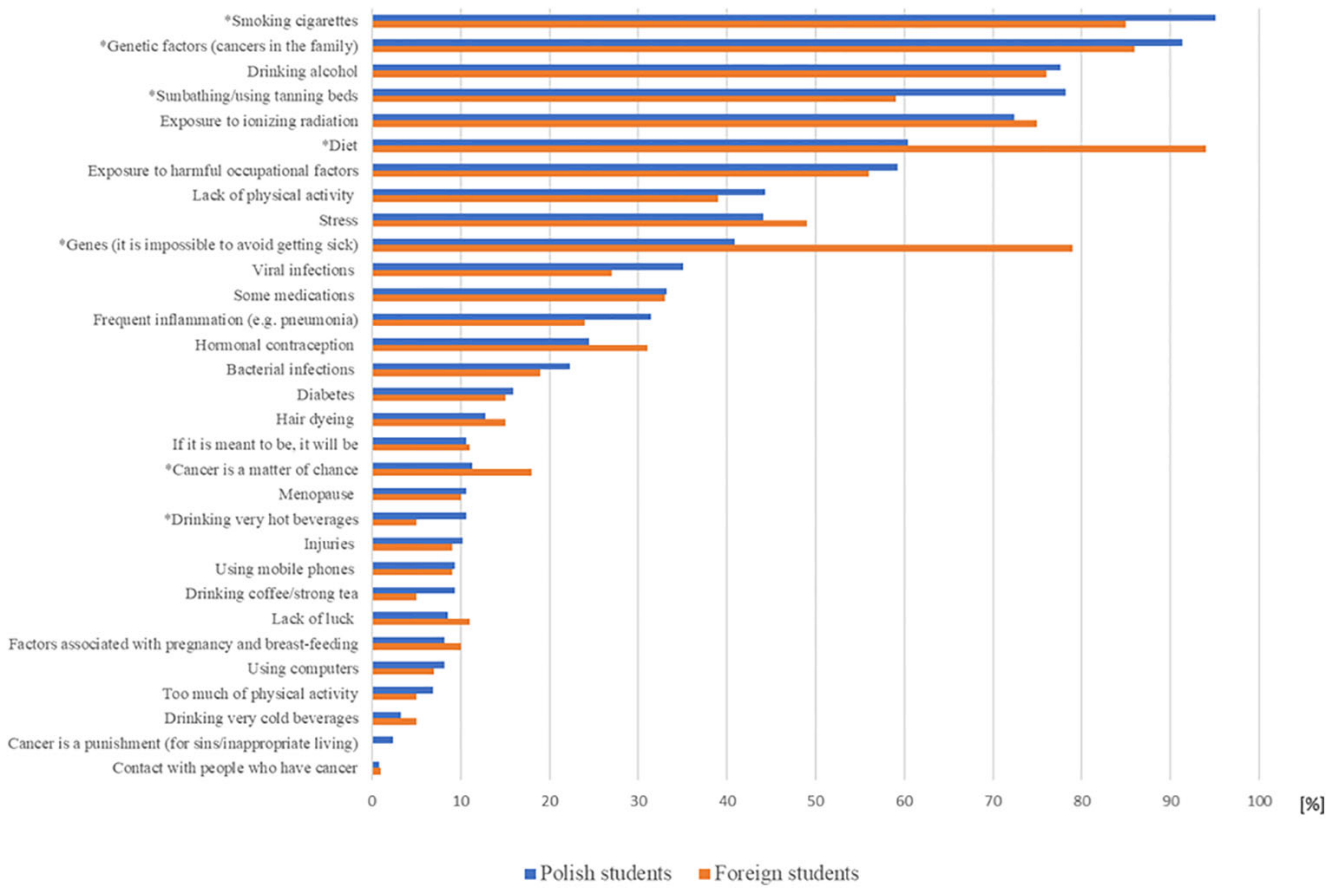
Potential cancer risk factors indicated by Polish and foreign students. *Significant difference between subgroups.

Almost half of students, both Polish and foreign, indicated that there is a correlation between age and cancer incidence (48% and 50%, respectively). Eighty-five percent of Polish students and 62% of international students recognized that cancer can be a hereditary disease (p < 0.001). About 90% of Polish and other students indicated that the presence of cancer in the family increases the risk of cancer. As potential risk factors of cancer development, both Polish and foreign students most commonly indicated smoking cigarettes, drinking alcohol, sunbathing/using tanning beds, exposure to ionizing radiation, diet, and exposure to harmful occupational factors. However, there were some discrepancies. Polish students were more likely than foreign students to identify smoking cigarettes (95% vs. 85%; p < 0.001) and sunbathing/using tanning beds (78% vs. 59%; p < 0.001) as risk factors, whereas foreign students more frequently identified diet as more significant factor compared to Polish students (94% vs. 60%; p < 0.001). Less frequently, students indicated lack of physical activity, viral infection, and hormonal contraception as potential risk factors (**Figure 1**).

Among the “three major cancer factors,” both Polish and foreign students, most frequently indicated genetic factors (cancers in the family) (81% vs. 75%; p = 0.13) and smoking cigarettes (70% vs. 56%; p = 0.001). Among the “three major cancer factors,” Polish students also pointed sunbathing/using tanning beds, and foreign students pointed exposure to ionizing radiation.

Polish students (86%) as well as international students (84%) reported that it is possible to reduce the incidence of cancer. Most participants responded that it is possible to cure cancer. However, significantly more Polish students than foreign students held this view (88% vs. 76%; p < 0.001).

**Figure 2:**
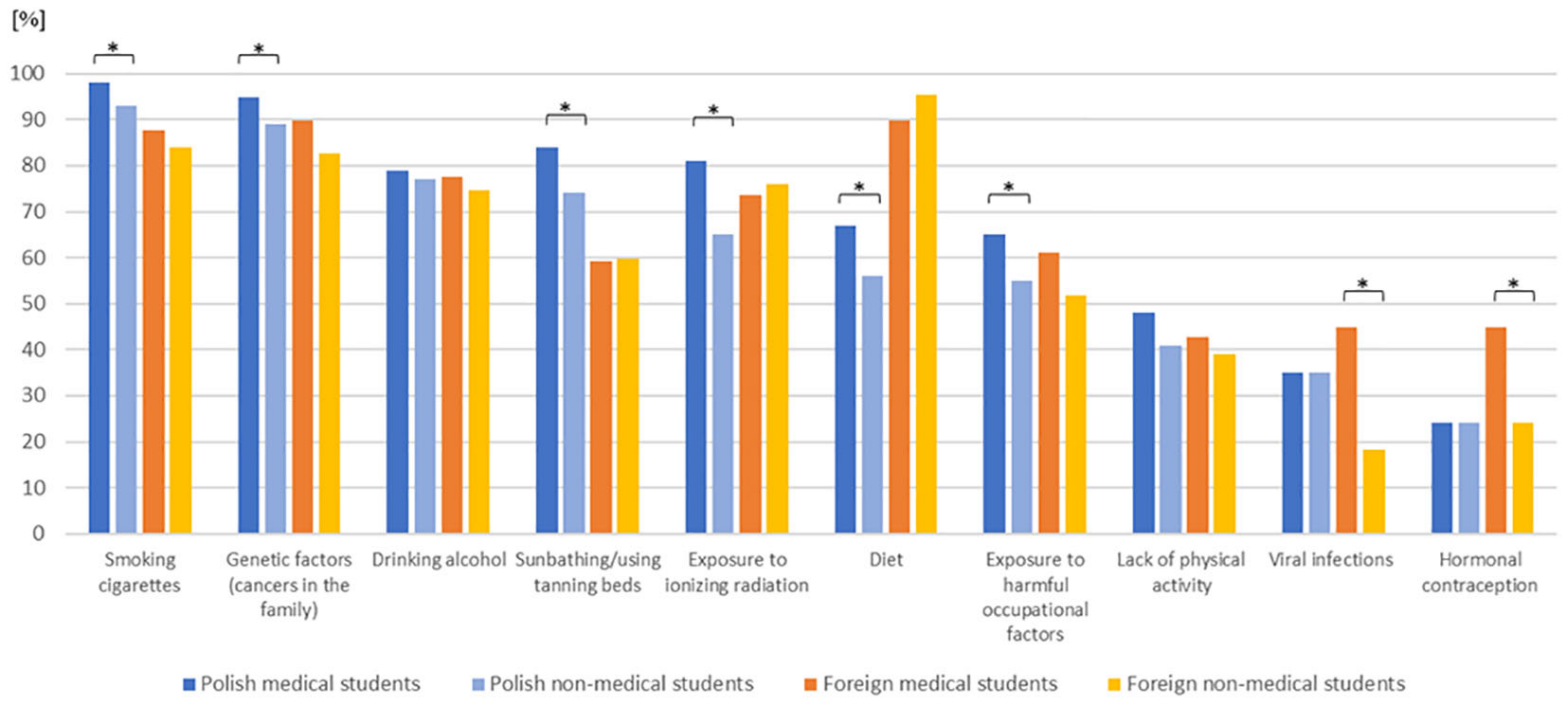
Potential cancer risk factors indicated by medical vs. non-medical Polish and foreign students. *Significant difference between subgroups.

Medical students demonstrated a superior knowledge regarding cancer risk factors compared with non-medical students. Among Polish students, significant differences were observed between the two groups with respect to the following factors: smoking cigarettes (98% vs. 93%; p = 0.002), genetic factors (cancers in the family) (95% vs. 89%; p = 0.007), sunbathing/using tanning beds (84% vs. 74%; p < 0.001), exposure to ionizing radiation (81% vs. 65%, p < 0.001), diet (67% vs. 56%; p = 0.002), and exposure to harmful occupational factors (65% vs. 55%; p = 0.004). Among international students, medical students were significantly more likely than non-medical students to indicate the following cancer risk factors: viral infections (45% vs. 18%; p < 0.001) and hormonal contraception (45% vs. 24%; p = 0.012) (**Figure 2**).

### 3.3 Multivariate analysis of cancer risk factors

All cancer risk factors mostly indicated by students were analyzed. Independent predictors for those factors were field of study and/or nationality. The association of cigarette smoking with cancer was acknowledged with notably greater frequency by students from Poland than by those from other countries (OR, 3.46; p < 0.001) and medical students than non-medical students (OR, 2.49; p = 0.006). Women more frequently than men (OR, 1.96; p = 0.007) and medical students more frequently than non-medical students (OR, 1.8; p = 0.02) reported genetic factors. Students who had close family or friends with cancer showed higher accuracy in identifying alcohol consumption as a risk factor for the development of cancer in comparison to these who had not (OR, 1.48; p = 0.019). Sunbathing/using tanning beds was indicated more often by Polish students than foreign students (OR, 3.23; p < 0.001), women than men (OR, 1.65; p = 0.006), medical students than non-medical students (OR, 1.49; p = 0.02), and students living in bigger cities (OR, 1.86; p = 0.005). Medical students exhibited a heightened perception of ionizing radiation as a cancer risk factor compared to non-medical students (OR, 1.99; p < 0.001). A poor diet was identified as a cancer risk factor more often by foreign students than Polish students (OR, 9.98; p < 0.001) and medical students than non-medical students (OR, 1.58; p = 0.002). Exposure to harmful occupational factors was pointed more frequently by Polish students than other students (OR, 1.55; p = 0.033), medical students than non-medical students (OR, 1.46; p = 0.008), and students residing in larger cities (OR, 1.82; p = 0.001) (**Table 2**).

**Table 2:** Multivariate analysis of beliefs about cancer risk factors among students worldwide.

Dependent variable	Significant variables	OR	95% CI	*p*
Cancer risk factors				
Smoking cigarettes				
	Nationality (foreign students)	Ref.		
	Nationality (Polish students)	3.46	(1.90–6.30)	<0.001
	Field of study (non-medical)	Ref.		
	Field of study (medical)	2.49	(1.30–4.76)	0.006
Genetic factors (cancers in the family)				
	Nationality (foreign students)	Ref.		
	Nationality (Polish students)	1.77	(1.01–3.08)	0.045
	Gender (men)	Ref.		
	Gender (women)	1.96	(1.20–3.20)	0.007
	Gender (other)	1.08	(0.13–9.17)	0.945
	Field of study (non-medical)	Ref.		
	Field of study (medical)	1.80	(1.08–3.00)	0.02
Drinking alcohol				
	Having cancer patient as a close friend/family (no)	Ref.		
	Having cancer patient as a close friend/family (yes)	1.48	(1.07–2.06)	0.019
Sunbathing/using tanning beds				
	Nationality (foreign students)	Ref.		
	Nationality (Polish students)	3.23	(2.08–5.01)	<0.001
	Gender (men)	Ref.		
	Gender (women)	1.65	(1.16–2.36)	0.006
	Gender (other)	1.16	(0.22–6.08)	0.86
	Field of study (non-medical)	Ref.		
	Field of study (medical)	1.49	(1.06–2.08)	0.020
	Place of residence (village)	Ref.		
	Place of residence (cities with <50,000 inhabitants)	0.81	(0.51–1.28)	0.368
	Place of residence (cities with 50,000–100,000 inhabitants)	0.57	(0.33–0.98)	0.042
	Place of residence (cities with >100,000 inhabitants)	1.86	(1.20–2.87)	0.005
Exposure to ionizing radiation				
	Field of study (non-medical)	Ref.		
	Field of study (medical)	1.99	(1.46–2.73)	<0.001
Diet				
	Nationality (Polish students)	Ref.		
	Nationality (foreign students)	9.98	(4.98–19.99)	<0.001
	Field of study (non-medical)	Ref.		
	Field of study (medical)	1.58	(1.18–2.12)	0.002
Exposure to harmful occupational factors				
	Nationality (foreign students)	Ref.		
	Nationality (Polish students)	1.55	(1.04-2.31)	0.033
	Field of study (non-medical)	Ref.		
	Field of study (medical)	1.46	(1.10-1.93)	0.008
	Place of residence (village)	Ref.		
	Place of residence (cities with <50,000 inhabitants)	0.80	(0.54-1.19)	0.270
	Place of residence (cities with 50,000–100,000 inhabitants)	1.02	(0.62-1.69)	0.93
	Place of residence (cities with >100,000 inhabitants)	1.82	(1.28-2.59)	0.001

Ref., Reference value (1.00).

## 4 Discussion

Most of the students in current study knew that cancers can have a genetic basis and that a family history of cancer increases the risk of developing cancer. Students most often named properly smoking, drinking alcohol, sunbathing/using tanning beds, exposure to ionizing radiation, diet, and exposure to harmful occupational agents as cancer risk factors. However, only less than half of the students pointed to other important factors, such as lack of physical activity, viral or bacterial infections, hormonal factors, and drinking hot beverages.

In fact, the causes of cancer are complex. There are known hereditary cancers and those that develop due to external factors. Many cancers depend on smoking, alcohol consumption, diet, physical activity, exposure to harmful factors such as sunlight, ionizing radiation, and occupational and infectious factors. Young people’s knowledge of the main causes of cancer is key to future reductions in cancer incidence and mortality in the population. Bad lifestyle habits such as smoking, drinking alcohol, using tanning bed, poor physical activity, and unhealthy eating habits have just been reported frequently among adolescents (18–22).

Cigarette smoking as a cancer-causing factor was mentioned by almost all students (95% of Polish and 85% of foreign students). Similarly, in the US in the study of adolescents’ perceptions of cancer risk factors among middle and high school students, 91% of participants recognized smoking as a behaviors affecting cancer risk factor (22). But among the “three major cancer factors,” 70% of Polish students and only 56% of foreign students indicated smoking cigarettes. In fact, smoking causes over 90% of lung cancer and up to 30% of all cancers in the developed countries (23, 24). In the previous study among Polish high school students aged 17–18 years, as a major cause of cancer, only 18.5% of students indicated smoking (25). However, in some studies, both adults and adolescence indicated smoking at the top of cancer risk factor (26–29).

The most of Polish students (91%) and foreign students (86%) pointed genetic factors as a cancer risk factor and they included it into the “three major cancer factors” (81% of Polish and 75% of foreign students). Study among even younger high school students reported that most of them perceived the genetic factors as the most important factors affecting cancer development (25). The study conducted by Karadeniz et al. (29) reported that 83% of adults indicated family history of cancer as a main risk factor for cancer.

In England, more than half of adults surveyed associated alcohol with cancer (30). Unfortunately, young Polish high school students from our previous work completely failed to see the link between drinking alcohol and cancer (25). Drinking of alcohol was recognized as a cancer risk factor by nearly 80% of Polish and foreign students in current study. Participants who reported that their relatives or friends had been diagnosed with cancer were significantly more likely to cite drinking alcohol as a risk factor for cancer. Similarly Lykins et al. (31) showed that, if there was cancer in family, then people significantly often pointed alcohol.

A significant difference was found in the responses of Polish and foreign students regarding whether diet plays a role in cancer development. There were a higher number of foreign students that believed so, compared to Polish students (94% vs. 60%). In the US, 80% of adolescents’ perceptions recognized poor diet as a cancer risk factor (22). It should be mentioned that Polish cuisine is rich in fats, especially saturated and refined carbohydrates, which can increase the risk of obesity (32). Especially since in 2022, Poland had the second highest prevalence of obesity in men among European countries (33). The study by Boylan et al. (34) showed that Polish people revealed a limited knowledge about healthy eating. Traditional Polish cuisine is still used because of the culture and conservative customs celebrated through food. Factors that were overlooked by respondents are infections. Viral infections were pointed by only 30% of students and bacterial infection by only 20% of students. Actually, approximately 13% of worldwide cancer diagnoses were associated with carcinogenic infections, mostly *Helicobacter pylori*, human papillomavirus (HPV), hepatitis B and C virus, and Epstein–Barr virus (14). In some countries (Belgium, Spain, UK), 85%–90% of students link HPV infection to cervical cancer, whereas only 20%–50% of students in others (Nigeria, Costa Rica, Scotland, and Turkey) (35–39). The discrepancy between different countries may be related to cultural attitudes toward health, the global population’s knowledge of cancer, encouragement of cancer screening, and the organization of health care. Eighty-five percent of all investigated students believe that cancer can be prevented. On the other hand, 41% of Polish students and as many as 79% of other students consider cancer to be an inherited disease and thus one that cannot be prevented. Our previous study showed that 81% of high school students claimed that people can modify their own cancer risk (35). In the US, 66% of adolescence indicated that individuals have control over their risk of cancer (22). Two-thirds of older adults (65 years and older) in Poland believed that cancer depends on their lifestyle and environment (40). Factors contributing to an elevated cancer risk can be categorized into intrinsic elements, which are unmodifiable, such as random mutations during DNA replication, and non-intrinsic factors, which can be divided into endogenous and exogenous categories. Endogenous factors are partially modifiable and include aspects like biological aging, genetic predisposition, and hormonal imbalances, whereas exogenous factors are fully modifiable and encompass elements such as radiation exposure, viral infections, unhealthy lifestyle choices, and occupational risks, which all could be avoided (41). It is crucial to recognize that students lack sufficient awareness regarding effective cancer precautionary measures and their application (42–44). Despite the correlation of students’ beliefs with risk factors and their influence on the development of the disease, they overestimated the importance of genetic predisposition.

By categorizing the student cohort from Poland and other countries into medical and non-medical, notable distinctions were identified in recognition of cigarette smoking, genetic factors (cancers in the family), sunbathing/using tanning beds, exposure to ionizing radiation, exposure to harmful occupational factors, and diet in favor of Polish medical students. Opposite foreign medical students more often indicated viral infections and hormonal contraception as important cancer factors in compare with non-medical students. However, viral and bacterial infections and lack of physical activity were relatively rare indicated by medical students—both Polish and foreign. This is in line with the results of other studies with research indicating that medical students do not have enough knowledge about cancer risk factors, despite their education (15, 42–45).

It is interesting that 18% of foreign students and 11% of Polish students indicated answer “cancer is a matter of chance.” Fatalistic beliefs about cancer that “if it is meant to be, it will be” and “lack of luck” showed about 10% both Polish and foreign students. In another study on Polish people but older (65 years and over), only 3% claimed that what “will happen will happen” (40). However, 24% of respondents considered that “if in family someone has a cancer, I will certainly get sick as well” (40). The fatalistic beliefs could effect on avoiding visits to physicians and participation in cancer screening.

### 4.1 Strengths and limitations of the study

There is a lack of studies on beliefs about cancer, especially among young people. The current study was conducted on a wide sample (almost one thousand students). The analysis was done in several countries. The discrepancy between number of Polish and foreign students was a limitation of the study. There was a domination of Indian, Swedish and Italian, except Polish students. Female predominance was also a limitation of this study (women are probably more likely to fill out this type of survey).

### 4.2 Application

Based on that, it seems reasonable to suggest introducing more educational programs focused on lifestyle changes. Young people, such as students, should have knowledge of cancer risk factors, because the earlier reduction of carcinogens causes the lower the risk of cancer. Probably, the right time to introduce education about health, including cancer risk factors, is high school. The results of this study can be used as an impulse for educational policy makers to integrate health education into high school curricula.

## 5 Conclusion

Students were able to correctly indicate the main cancer risk factors, such as smoking, drinking alcohol, UV radiation, and diet. They overestimated the importance of genetic factors while not recognizing some of the important modifiable factors, such as lack of physical activity or viral infections. Furthermore, Polish students were less knowledgeable on nutrition than students from other countries. Medical students had a better understanding of cancer risk factors in comparison with non-medical students.

## Data Availability

The raw data supporting the conclusions of this article will be made available by the authors, without undue reservation.
