# Supplementary material for: Beliefs about causes of cancer among students around the world
Source: Front Oncol. 2025 Aug 1;15:1631997. doi: 10.3389/fonc.2025.1631997 (PMC12353720; doi:10.3389/fonc.2025.1631997)
Supplement: Supplementary file 1 [file DataSheet1.docx]

Dear Colleagues,

We are members of the Epidemiological Research Scientific Student Club active at the Medical Faculty of University of Warmia and Mazury in Olsztyn.

We would like to invite you to take part in the research, which is directed to students of all fields from different countries. The aim of the study is to assess beliefs about causes of cancer.

Participation in the study is voluntary and anonymous. The collected data will be used to write a manuscript for publishing in a scientific journal.

By starting to fill in the questionnaire, you give your consent to participate in the study.

Thank you for your time.

1. Age …….years old

2. Gender

- Female
- Male
- Other

3. Country of residence…..

4. Place of residence:

- Village
- City <50,000 inhabitants
- City 50,000-100,000 inhabitants
- City >100,000 inhabitants

5. Nationality………………….

6. Field of study…………………

7. Year of study………………..

8. Do you have a person diagnosed with cancer in close family or friends?

- Yes
- No
- I do not know

9. Is there a correlation between age and cancer incidence?

- Yes
- No
- I do not know

10. Are there hereditary cancers?

- Yes
- No
- I do not know

11. Which of the following factors are associated with cancer? (you can choose more than one)

- Genetic factors (cancers in the family)
- Genes (it is impossible to avoid getting sick)
- Diet
- Drinking very hot beverages
- Drinking very cold beverages
- Smoking cigarettes
- Drinking alcohol
- Drinking coffee/strong tea
- Lack of physical activity
- Too much of physical activity
- Exposure to harmful occupational factors
- Exposure to ionizing radiation
- Sunbathing/using tanning beds
- Hair dyeing
- Injuries
- Viral infections
- Bacterial infections
- Some medications
- Using mobile phones
- Using computers
- Diabetes
- Frequent inflammation (e.g. pneumonia)
- Factors associated with pregnancy and breast-feeding
- Menopause
- Hormonal contraception
- Stress
- Contact with people who have cancer
- Cancer is a matter of chance
- Cancer is a punishment (for sins/inappropriate living)
- If it is meant to be, it will be
- It is lack of luck

12. What are the main factors causing cancer? (please select maximum 3 answers)

- Genetic factors (cancers in the family)
- Genes (it is impossible to avoid getting sick)
- Diet
- Drinking very hot beverages
- Drinking very cold beverages
- Smoking cigarettes
- Drinking alcohol
- Drinking coffee/strong tea
- Lack of physical activity
- Too much of physical activity
- Exposure to harmful occupational factors
- Exposure to ionizing radiation
- Sunbathing/using tanning beds
- Hair dyeing
- Injuries
- Viral infections
- Bacterial infections
- Some medications
- Using mobile phones
- Using computers
- Diabetes
- Frequent inflammation (e.g. pneumonia)
- Factors associated with pregnancy and breast-feeding
- Menopause
- Hormonal contraception
- Stress
- Contact with people who have cancer
- Cancer is a matter of chance
- Cancer is a punishment (for sins/inappropriate living)
- If it is meant to be, it will be
- It is lack of luck

13. Is there any way to reduce the risk of cancer?

- Yes
- No
- I do not know

14. Can cancer be cured?

- Yes
- No
- I do not know
