# Supplementary material for: Beliefs about causes of cancer among students around the world
Source: Front Oncol. 2025 Aug 1;15:1631997. doi: 10.3389/fonc.2025.1631997 (PMC12353720; doi:10.3389/fonc.2025.1631997)
Supplement: Supplementary file 2 [file DataSheet2.docx]

Drodzy Koledzy,

Jesteśmy studentami z Koła Naukowego Badań Epidemiologicznych Uniwersytetu Warmińsko-Mazurskiego w Olsztynie.

Chcielibyśmy zaprosić Was do wzięcia udziału w badaniu ankietowym, którego celem jest ocena przekonań związanych z przyczynami raka.

Badanie skierowane jest do studentów wszystkich kierunków z różnych krajów.

Udział w badaniu jest dobrowolny i anonimowy. Zgromadzone dane zostaną wykorzystane do napisania pracy naukowej.

Rozpoczęcie wypełniania ankiety jest jednoznaczne z wyrażeniem zgody na udział w badaniu.

Dziękujemy za poświęcony czas.

1. Wiek…….lat

2. Płeć

- Kobieta
- Mężczyzna
- Inne

3. Kraj zamieszkania…..

4. Miejsce zamieszkania

- Wieś
- Miasto do 50 tysięcy mieszkańców
- Miasto 50-100 tysięcy mieszkańców
- Miasto powyżej 100 tysięcy mieszkańców

5. Narodowość ……………..

6. Kierunek studiów…………..

7. Rok studiów…………..

8. Czy ktoś z Twojej najbliższej rodziny lub przyjaciół chorował na nowotwór?

- Tak
- Nie
- Nie wiem

9. Czy występowanie nowotworów ma związek z wiekiem?

- Tak
- Nie
- Nie wiem

10. Czy nowotwory mogą być chorobami dziedzicznymi?

- Tak
- Nie
- Nie wiem

11. Z czym związane jest powstanie nowotworu? (można zaznaczyć wiele odpowiedzi)

- Czynnik genetyczny (nowotwory w rodzinie)
- Geny (nie da się uniknąć zachorowania)
- Nieodpowiednia dieta
- Picie bardzo gorących napojów
- Picie bardzo zimnych napojów
- Palenie papierosów
- Picie alkoholu
- Picie kawy/mocnej herbaty
- Brak aktywności fizycznej
- Zbyt intensywna aktywność fizyczna
- Narażenie na szkodliwe czynniki zawodowe
- Narażenie na promieniowanie jonizujące
- Opalanie się/korzystanie z łóżek opalających
- Farby do włosów
- Urazy
- Infekcje niektórymi wirusami
- Infekcje bakteryjne
- Niektóre leki
- Używanie telefonów komórkowych
- Używanie komputerów
- Cukrzyca
- Częste zapalenia (np. płuc)
- Czynniki związane z ciążą i karmieniem piersią
- Zmiany wywoływane przez menopauzę
- Antykoncepcja hormonalna
- Stres
- Kontakt z innymi osobami chorymi na raka
- Rak to kwestia przypadku
- Rak jest karą (za grzechy/nieodpowiednie życie)
- Jak ma być to i tak będzie
- Jest kwestią braku szczęścia

12. Co jest głównymi czynnikami wywołującymi nowotwory? (proszę zaznaczyć maksymalnie 3 odpowiedzi)

- Czynnik genetyczny (nowotwory w rodzinie)
- Geny (nie da się uniknąć zachorowania)
- Nieodpowiednia dieta
- Picie bardzo gorących napojów
- Picie bardzo zimnych napojów
- Palenie papierosów
- Picie alkoholu
- Picie kawy/mocnej herbaty
- Brak aktywności fizycznej
- Zbyt intensywna aktywność fizyczna
- Narażenie na szkodliwe czynniki zawodowe
- Narażenie na promieniowanie jonizujące
- Opalanie się/korzystanie z łóżek opalających
- Farby do włosów
- Urazy
- Infekcje niektórymi wirusami
- Infekcje bakteryjne
- Niektóre leki
- Używanie telefonów komórkowych
- Używanie komputerów
- Cukrzyca
- Częste zapalenia (np. płuc)
- Czynniki związane z ciążą i karmieniem piersią
- Zmiany wywoływane przez menopauzę
- Antykoncepcja hormonalna
- Stres
- Kontakt z innymi osobami chorymi na raka
- Rak to kwestia przypadku
- Rak jest karą (za grzechy/nieodpowiednie życie)
- Jak ma być to i tak będzie
- Jest kwestią braku szczęścia

13. Czy można w jakiś sposób zmniejszyć ryzyko zachorowania na nowotwór?

- Tak
- Nie
- Nie wiem

14. Czy można wyleczyć chorego na nowotwór?

- Tak
- Nie
- Nie wiem
