# Supplementary material for: Beliefs about causes of cancer among students around the world
Source: Front Oncol. 2025 Aug 1;15:1631997. doi: 10.3389/fonc.2025.1631997 (PMC12353720; doi:10.3389/fonc.2025.1631997)
Supplement: Supplementary file 3 [file Table1.docx]

Table A. Nationality of foreign students

| Foreign students' group | n=140 | % |
| --- | --- | --- |
| Indian | 26 | 18.57 |
| Swedish | 12 | 8.57 |
| Italian | 12 | 8.57 |
| Pakistani | 10 | 7.14 |
| Spanish | 8 | 5.71 |
| Norwegian | 7 | 5.00 |
| German | 7 | 5.00 |
| Iraqi | 7 | 5.00 |
| Mexican | 7 | 5.00 |
| French | 6 | 4.29 |
| Japanese | 4 | 2.86 |
| Turkish | 4 | 2.86 |
| Romanian | 3 | 2.14 |
| Filipino | 3 | 2.14 |
| Dannish | 3 | 2.14 |
| Jamaican | 2 | 1.43 |
| Zimbabwean | 2 | 1.43 |
| Afghan | 2 | 1.43 |
| Nigerian | 2 | 1.43 |
| Bangladeshi | 1 | 0.71 |
| Rwandese | 1 | 0.71 |
| Egyptian | 1 | 0.71 |
| Lebanese | 1 | 0.71 |
| Kenyan | 1 | 0.71 |
| Chinese | 1 | 0.71 |
| Singaporean | 1 | 0.71 |
| Malaysian | 1 | 0.71 |
| American | 1 | 0.71 |
| Armenian | 1 | 0.71 |
| Eritrean | 1 | 0.71 |
| Ukrainian | 1 | 0.71 |
| Albanian | 1 | 0.71 |
